# Association between lipid variability and the risk of mortality in cancer patients not receiving lipid-lowering agents

**DOI:** 10.3389/fonc.2023.1254339

**Published:** 2023-10-05

**Authors:** Seohyun Kim, Gyuri Kim, So Hyun Cho, Rosa Oh, Ji Yoon Kim, You-Bin Lee, Sang-Man Jin, Kyu Yeon Hur, Jae Hyeon Kim

**Affiliations:** ^1^Department of Clinical Research Design and Evaluation, Samsung Advanced Institute for Health Sciences and Technology, Sungkyunkwan University, Seoul, Republic of Korea; ^2^Division of Endocrinology and Metabolism, Department of Medicine, Samsung Medical Center, Sungkyunkwan University School of Medicine, Seoul, Republic of Korea

**Keywords:** total cholesterol, LDL cholesterol, variability, mortality, cancer

## Abstract

**Aim:**

We investigated the association between total cholesterol (TC), low-density lipoprotein (LDL) cholesterol, high-density lipoprotein (HDL) cholesterol, and triglyceride (TG) variability and cancer patient mortality risk.

**Methods:**

We retrospectively analyzed 42,539 cancer patients who were not receiving lipid-lowering agents and who had at least three TC measurements within 2 years of their initial cancer diagnosis. Using a multivariable Cox regression model, the risk of mortality was evaluated.

**Results:**

In multivariable analysis, Q2 (adjusted hazard ratio [aHR]: 1.32, 95% confidence interval (CI): 1.24–1.41), Q3 (aHR: 1.66, 95% CI: 1.56–1.76), and Q4 (aHR: 1.96, 95% CI: 1.84–2.08) of coefficient of variation (CV) in TC were significantly associated with mortality risk compared to Q1, showing a linear association between higher TC variability and mortality (*P* for trend<0.001). Q2 (aHR: 1.34, 95% CI: 1.06–1.77), Q3 (aHR: 1.40, 95% CI: 1.06–1.85), and Q4 (aHR: 1.50, 95% CI: 1.14–1.97) were all significantly associated with a higher risk of death compared to Q1 in multivariable Cox regression for the association between CV in LDL and all-cause mortality (*P* for trend=0.005).

**Conclusion:**

In cancer patients who do not receive lipid-lowering agents, high variability in total cholesterol and LDL cholesterol levels was found to pose significant role in mortality risk.

## 1 Introduction

In 2015, cancer was the second-leading cause of death globally after cardiovascular diseases, accounting for approximately 8.7 million deaths (1). In 20 regions of the world, 18.1 million new cancer cases (excluding non-melanoma skin cancer) and 9.6 million cancer-related deaths (9.5 million excluding non-melanoma skin cancer) were anticipated in 2018 (2). In addition to physicians’ efforts to stratify risk factors for cancer treatment based on the TNM staging system, new prognostic biomarkers that may help stratify cancer risk and modify risk have been identified (3). Cancer patients had significant associations between total cholesterol (TC) and high-density lipoprotein cholesterol (HDL-C) levels and overall survival, according to a systematic review and meta-analysis (4).

In the Framingham Heart Study, cholesterol variability was associated with mortality. This relationship has also been observed in recent studies of general populations and people with type 2 diabetes mellitus (DM) (5–8). Coronary atheroma progression, endothelial dysfunction, and the effect of many drugs on cholesterol levels were among the possible mechanisms of the effect of cholesterol variability on poor outcomes (6, 7, 9–11). Previous studies reported no significant association between intra-individual variability in TC levels stratified by quartile and cancer mortality (12). However, significant associations were found in all-cause and cardiovascular disease mortality (12).

To the best of our knowledge, although the association between cholesterol variability and mortality risk in cancer patients was evaluated in subgroup analysis in a previous study, no study examined the association between lipid variability and mortality risk in cancer patients who did not receive lipid-lowering agents in a large cohort. Therefore, we aimed to investigate the association between TC variability and mortality in newly diagnosed cancer patients not receiving lipid-lowering agents at the time of cancer diagnosis. Furthermore, we investigated the association between variability in low-density lipoprotein cholesterol (LDL-C), high-density lipoprotein cholesterol (HDL-C), and triglyceride (TG) and death in cancer patients not receiving lipid-lowering agents at the time of cancer diagnosis.

## 2 Materials and methods

### 2.1 Study design and population

**Figure 1:**
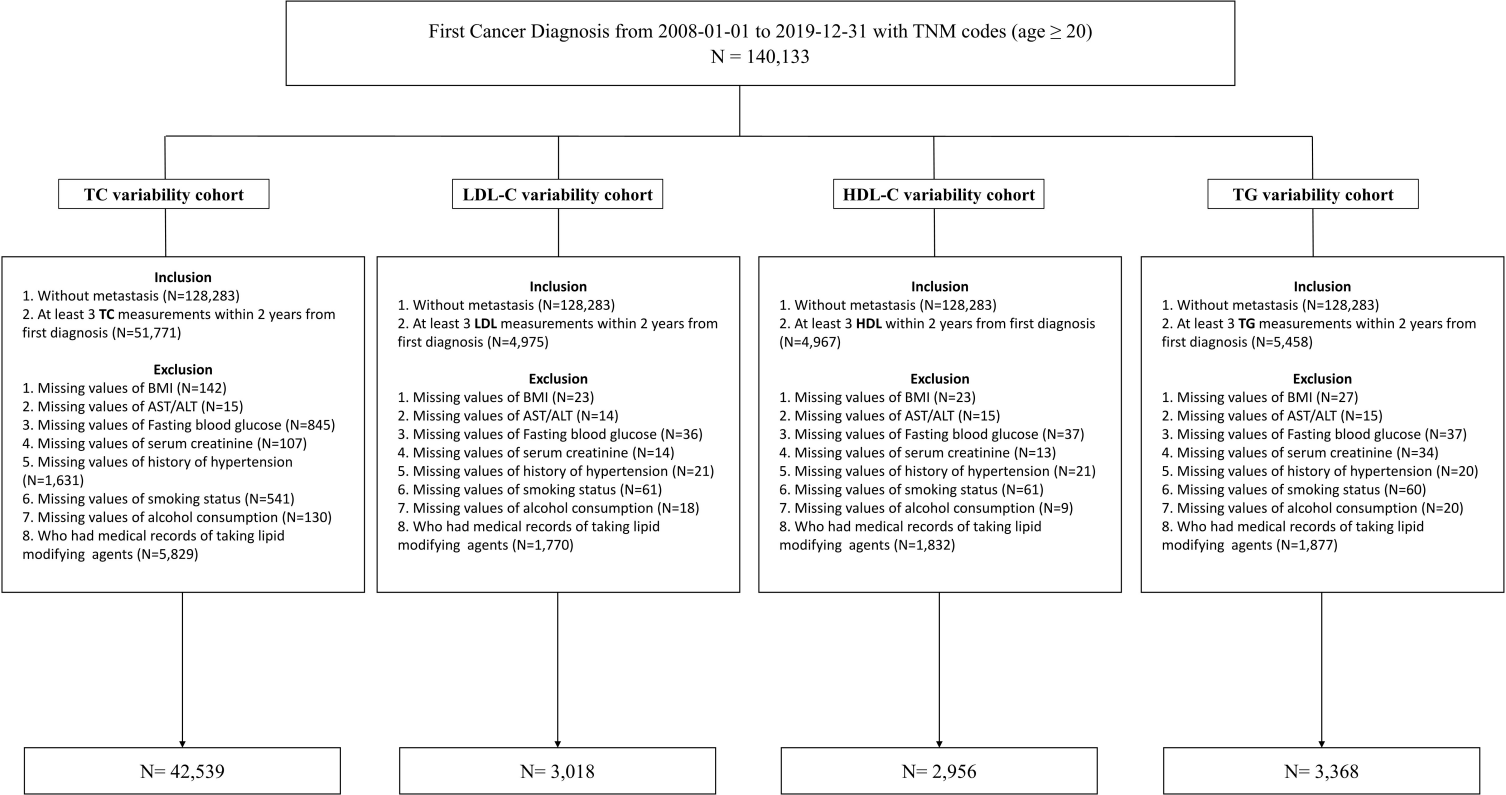
Flow chart of the study.

The study’s participants were newly diagnosed cancer patients older than 20 who visited the Samsung Medical Center (SMC) in Seoul, Republic of Korea, between January 2008 and December 2019. The study included 140,133 individuals diagnosed with cancer for the first time (International Classification of Disease, 10th revision (ICD-10), C code) and had medical records, including the TNM stage. We included cancer patients without metastasis who had at least three TC measurements within 2 years of their initial diagnosis (N=51,771). We excluded patients whose body mass index (BMI), laboratory measurements, medical history of hypertension (HTN), smoking status, and alcohol consumption variables were missing (N=3,411) and who had a history of prescriptions for lipid-lowering agents within a year from the index date (n=7,099). The LDL-C, HDL-C, and TG analysis datasets were created in the same process described previously. The workflow of the study is summarized at **Figure 1**. Finally, 42,539, 3,018, 2,956, and 3,368 participants were analyzed in the TC cohort, LDL-C cohort, HDL-C cohort, and TG cohort, respectively. All data were extracted from SMC’s clinical data warehouse (CDW), DARWIN-C.

### 2.2 Definition of exposure

After an overnight fast, laboratory measurements of the TC, LDL-C, HDL-C, and TG measured repeatedly from the patient’s first cancer diagnosis for two years were collected longitudinally. Mean and standard deviation (SD) values were calculated from blood lipid levels measured at least thrice within 2 years of the initial diagnosis for each cancer patient. We calculated the mean value and standard deviation from repeatedly measured cholesterol levels, and the coefficient of variation (CV) in TC, LDL-C, HDL-C, and TG was defined as the ratio of the SD to the mean in each patient. And the CV is divided by the quartile, and the CV increases from the first quartile to the fourth quartile. Each cancer patient’s last blood lipid measurement date was defined as the index date.

### 2.3 Definition of covariates

Electrical medical records and a self-administered questionnaire were used to examine the personal medical history, including DM, HTN, smoking history, alcohol intake, and medications. The demographic, anthropometric, and laboratory data of all participants were collected. ICD-10 codes E11–14, a self-reported history of DM, records of anti-diabetic drug prescriptions, an HbA1c of 6.5% or above, or a fasting glucose level of 126 mg/dL or higher were used to identify DM. The chronic kidney disease epidemiology collaboration 2021 (CKD-EPI 2021) formula was used to calculate the value of the estimated glomerular filtration rate (eGFR) (13). We defined chronic kidney disease (CKD) as eGFR<60 mL/min/1.73 m^2^ at the index date (14). BMI was calculated by dividing body weight (kg) by height squared (m^2^). Information on alcohol intake and smoking status was collected by a self-reported questionnaire and classified as never, previous, or current. A history of HTN was indicated by the presence of I10–15 in ICD-10 codes, records of prescriptions for anti-hypertensive medications, a self-reported history of HTN, or systolic or diastolic blood pressure readings of >140 mmHg or >90 mmHg, respectively, measured at least three times. Medical history of all HMG-CoA reductase inhibitors, fenofibrate, bezafibrate, omega-3 acids, and self-report of medication use were all included in the lipid-lowering medications. All cancers were divided into 24 common categories based on the primary site of the disease and then reclassified into eight cancer types, including gastrointestinal (colon, rectum, stomach, esophagus, and small intestine), urologic (bladder, prostate, testis, and ureter), gynecologic (endometrial, cervix uteri, corpus uteri, and ovary), breast, hepato-pancreatobiliary (liver and intrahepatic bile duct, gallbladder and other parts of the biliary tract, and pancreas), lung, thyroid cancer, and other cancers (15–17).

### 2.4 Definition of outcomes

All participants were followed from the index date until the conclusion of the research (December 2020) or until their death (collected from the death records of SMC CDW linked to Statistics Korea).

### 2.5 Statistical analysis

All categorical variables were presented as percentages, while all continuous variables were shown as the mean and standard deviation (SD). The analysis of variance (ANOVA) for continuous variables and the chi-square test for categorical values were utilized to evaluate the characteristics of patients according to the CV quartile in lipid measurements. Kaplan–Meier’s method was used to examine survival curves, and the results were compared with the log-rank test. We estimated hazard ratios (HRs) with a 95% confidence interval (CI) for all-cause mortality using Cox proportional hazard regression models. We fitted three models to examine the association between variability in lipid measurements and death in cancer patients not receiving lipid-lowering agents. Model 1 was a crude model. Model 2 was adjusted for age, sex, BMI, and mean lipid measurements. Model 3 was further adjusted for CKD, DM, HTN, smoking status, alcohol consumption, and cancer type. We conducted a sensitivity analysis according to no-evidence-of-disease (NED) status and the administration of lipid-lowering agents during the follow-up study period. In sensitivity analyses, cancer patients were censored at the date of initiating NED status rather than continuing follow-up until the end of the study when they attained NED status, which the oncologists decided throughout follow-up (18). Furthermore, if lipid-lowering agents were administered during the study period, the follow-up was limited to the observation date. We performed subgroup analysis by age, sex, presence of DM and HTN, smoking status, alcohol consumption, and cancer type, and the highest quartile (Q4) group’s HR (95% CI) of CV in TC was compared with the lowest three quartiles (Q1–3) as a reference group. Additionally, we conducted an additional analysis to evaluate the association between the longitudinal absolute changes in TC from baseline and mortality using generalized estimating equation (GEE) models. R version 2.1.3 was used to perform all analyses, and *P<*0.05 was considered statistically significant.

## 3 Results

### 3.1 Baseline characteristics of the study population

**Table 1** shows all baseline characteristics stratified by the CV quartile in TC. Patients in higher quartiles of TC variability were older, more likely to be female, tended to have a lower BMI, and had a higher prevalence of DM and HTN. The CV values of TC in Q1, Q2, Q3, and Q4 were 5.7 ± 1.8, 10.0 ± 1.0, 13.9 ± 1.3, and 21.6 ± 4.8, respectively. The mean lipid values were highest in Q1 and lowest in Q4. The median frequency of total cholesterol measurement per patients is six times (25%tile: four times, 75%tile: nine times), while the median frequency of LDL-C, HDL-C, and TG is four times (25%tile: three times, 75%tile: five times). The median time interval for repeat TC measurement is 21 days (25%tile: four days, 75%tile: 68 days), while the median time interval for LDL-C, HDL-C, and TG is 91 days (25%tile: 35 days, 75%tile: 175 days).

**Table 1:** Baseline characteristics of the study participants according to the total cholesterol variability.

	Q1 (≤8.2) (N=10,632)	Q2 (8.2–11.8) (N=10,698)	Q3 (11.8–16.4) (N=10,623)	Q4 (>16.4) (N=10,586)	*P* value
Age	57.2 ± 11.5	57.1 ± 11.7	57.3 ± 11.9	59.0 ± 11.9	<0.001
Sex					<0.001
- Female	5479 (51.5%)	5259 (49.2%)	4687 (44.1%)	3874 (36.6%)	
- Male	5153 (48.5%)	5439 (50.8%)	5936 (55.9%)	6712 (63.4%)	
BMI	24.3 ± 16.0	24.5 ± 22.0	24.7 ± 27.0	23.7 ± 22.3	0.007
Serum creatinine	0.8 ± 0.4	0.8 ± 0.4	0.8 ± 0.5	0.9 ± 0.5	<0.001
Fasting glucose	105.7 ± 25.9	106.6 ± 27.5	107.5 ± 30.4	109.7 ± 34.4	<0.001
ALT	24.8 ± 26.2	24.1 ± 21.6	25.4 ± 45.5	26.6 ± 38.9	<0.001
AST	25.9 ± 25.5	25.7 ± 19.0	27.7 ± 52.8	29.5 ± 46.3	<0.001
eGFR	95.5 ± 16.0	95.6 ± 16.4	95.3 ± 17.4	94.0 ± 18.2	<0.001
Mean of lipid values	173.7 ± 31.9	169.1 ± 29.6	163.4 ± 28.6	154.0 ± 25.0	<0.001
SD of lipid values	9.9 ± 3.6	16.9 ± 3.4	22.8 ± 4.4	33.1 ± 8.2	<0.001
CV of lipid values	5.7 ± 1.8	10.0 ± 1.0	13.9 ± 1.3	21.6 ± 4.8	<0.001
Presence of CKD					<0.001
- No	10263 (96.5%)	10313 (96.4%)	10191 (95.9%)	10055 (95.0%)	
- Yes	369 (3.5%)	385 (3.6%)	432 (4.1%)	531 (5.0%)	
Presence of DM					<0.001
- No	8833 (83.1%)	8678 (81.1%)	8393 (79.0%)	8033 (75.9%)	
- Yes	1799 (16.9%)	2020 (18.9%)	2230 (21.0%)	2553 (24.1%)	
Presence of hypertension					<0.001
- No	6807 (64.0%)	6625 (61.9%)	6261 (58.9%)	5528 (52.2%)	
- Yes	3825 (36.0%)	4073 (38.1%)	4362 (41.1%)	5058 (47.8%)	
Smoking status					<0.001
- Never	7124 (67.0%)	6946 (64.9%)	6520 (61.4%)	6199 (58.6%)	
- Ever	1822 (17.1%)	1913 (17.9%)	2056 (19.4%)	2103 (19.9%)	
- Current	1686 (15.9%)	1839 (17.2%)	2047 (19.3%)	2284 (21.6%)	
Alcohol consumption					<0.001
- Never	6366 (59.9%)	6235 (58.3%)	5915 (55.7%)	5532 (52.3%)	
- Ever	2362 (22.2%)	2671 (25.0%)	2880 (27.1%)	3303 (31.2%)	
- Current	1904 (17.9%)	1792 (16.8%)	1828 (17.2%)	1751 (16.5%)	
Cancer types					<0.001
Gastrointestinal	3965 (37.3%)	4435 (41.5%)	4728 (44.5%)	5153 (48.7%)	
Urology	369 (3.5%)	217 (2.0%)	192 (1.8%)	183 (1.7%)	
Gynecology	39 (0.4%)	24 (0.2%)	27 (0.3%)	21 (0.2%)	
Breast	2386 (22.4%)	2326 (21.7%)	1825 (17.2%)	989 (9.3%)	
Hepato-Pancreatobiliary	666 (6.3%)	789 (7.4%)	1143 (10.8%)	1978 (18.7%)	
Lung	2176 (20.5%)	2167 (20.3%)	2077 (19.6%)	1490 (14.1%)	
Thyroid	381 (3.6%)	256 (2.4%)	174 (1.6%)	171 (1.6%)	
Others	650 (6.1%)	484 (4.5%)	457 (4.3%)	601 (5.7%)	

*ALT, alanine aminotransferase; AST, aspartate aminotransferase; BMI, body mass index; CKD, chronic kidney disease; CV, coefficient of variation; DM, diabetes mellitus; eGFR, estimated glomerular filtration rate; SD, standard deviation.

### 3.2 Multivariable Cox regression of all-cause mortality

**Figure 2:**
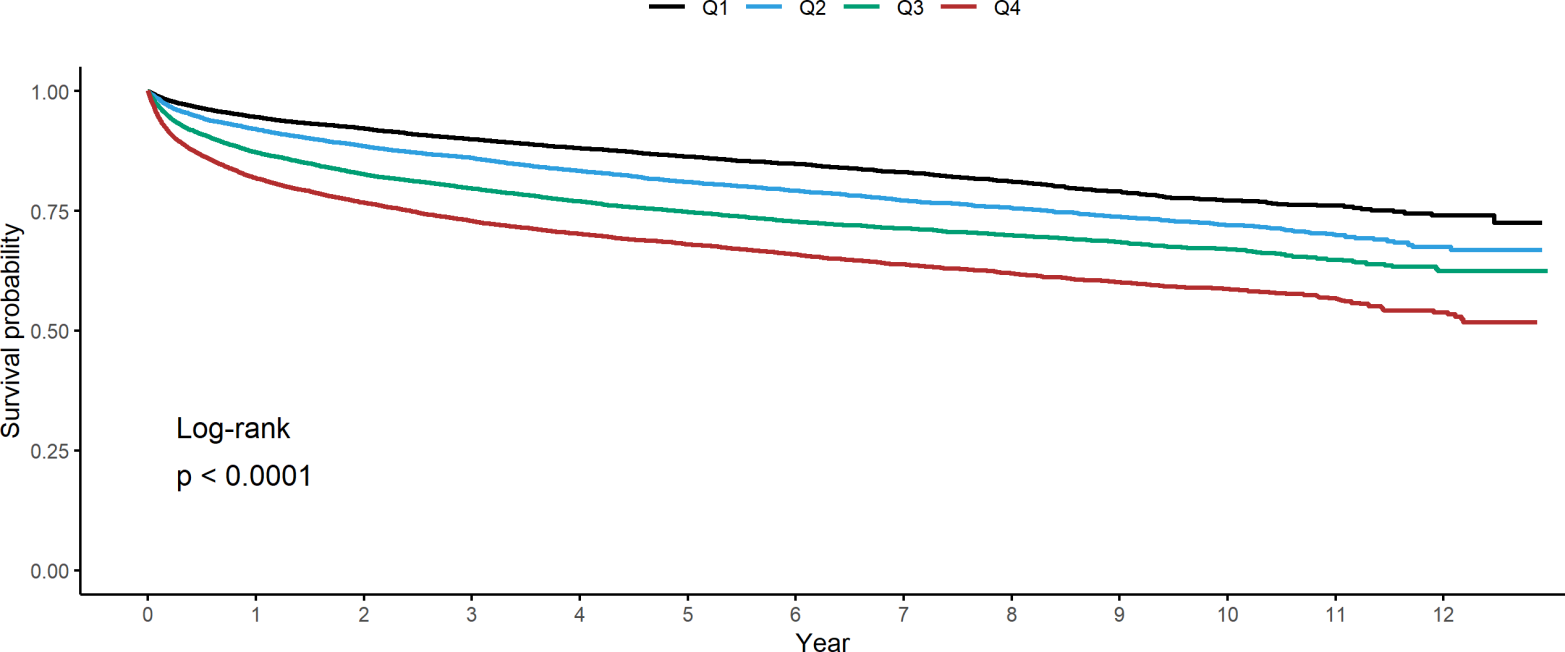
Kaplan-Meier estimates for all-cause mortality in cancer patients stratified by quartile of coefficient of variation in total cholesterol.

The median follow-up duration of all participants was 4.74 years (25%tile: 2.02 years, 75%tile: 7.64 years). Of the 10,239 deaths and 213,223 person-years, the incidence rate (per 10,000 person-years) for participants in Q1, Q2, Q3, and Q4 was 288.4, 394.9, 534.9, and 723.3, respectively. **Table 2** shows the result of a multivariable Cox regression of all-cause mortality with incidence rate. The unadjusted HRs for all-cause mortality in cancer patients with Q2, Q3, and Q4 of CV in TC were 1.38 (95% CI: 1.29–1.47), 1.86 (95% CI: 1.75–1.98), and 2.52 (95% CI: 2.37–2.67), compared to Q1 (**Figure 2**). In model 3 of multivariable analysis, Q2 (adjusted HR [aHR]: 1.32, 95% CI: 1.24–1.41), Q3 (aHR: 1.66, 95% CI: 1.56–1.76), and Q4 (aHR: 1.96, 95% CI: 1.84–2.08) were significantly associated with an increased risk of mortality compared to Q1 (*P* for trend<0.001). Q2 (aHR: 1.34, 95% CI: 1.06–1.77), Q3 (aHR: 1.40, 95% CI: 1.06–1.85), and Q4 (aHR: 1.50, 95% CI: 1.14–1.97) were all significantly associated with a higher risk of death compared to Q1 in multivariable Cox regression for the association between CV in LDL and all-cause mortality (*P* for trend=0.005). Only Q4 (aHR: 2.38, 95% CI: 1.78–3.18) was significantly associated with an increased risk of death relative to Q1 in multivariable Cox regression for the relationship between CV in HDL and all-cause mortality, but not Q2 or Q3 (*P* for trend<0.001). No association was observed between CV in TG and the risk of death (*P* for trend=0.722).

**Table 2:** Multivariable Cox regression for mortality by quartile of total cholesterol, LDL-C, HDL-C, and TG.

	Events	Person-years	Incidence Rate (per 10,000 py)	Model 1	Model 2	Model 3	P ^α^ for trend
Association between TC variability and overall survival							
Q1 (≤8.2) (N=10,632)	1582	54,847	288.4	ref	ref	ref	<0.001
Q2 (8.2–11.8) (N=10,698)	2171	54,977	394.9	1.38 (1.29–1.47)	1.33 (1.24–1.41)	1.32 (1.24–1.41)	
Q3 (11.8–16.4) (N=10,623)	2818	52,684	534.9	1.86 (1.75–1.98)	1.69 (1.59–1.80)	1.66 (1.56–1.76)	
Q4 (>16.4) (N=10,586)	3668	50,715	723.3	2.52 (2.37–2.67)	1.98 (1.87–2.11)	1.96 (1.84–2.08)	
Association between LDL variability and overall survival							
Q1 (≤8.7) (N=755)	88	2,658	331.1	ref	ref	ref	0.005
Q2 (8.7–13.2) (N=759)	117	2,591	451.5	1.36 (1.03–1.79)	1.35 (1.02–1.78)	1.34 (1.02–1.77)	
Q3 (13.2–18.9) (N=755)	115	2,352	489.0	1.43 (1.08–1.89)	1.40 (1.06–1.85)	1.40 (1.06–1.85)	
Q4 (>18.9) (N=749)	130	2,409	539.6	1.61 (1.23–2.11)	1.47 (1.12–1.94)	1.50 (1.14–1.97)	
Association between HDL variability and overall survival							
Q1 (≤8.4) (N=739)	69	2,777	248.5	ref	ref	ref	<0.001
Q2 (8.4–12.6) (N=733)	89	2,594	343.1	1.37 (1.00–1.87)	1.32 (0.96–1.81)	1.17 (0.85–1.61)	
Q3 (12.6–18.4) (N=742)	106	2,472	428.7	1.69 (1.24–2.28)	1.53 (1.13–2.08)	1.35 (1.00–1.84)	
Q4 (>18.4) (N=742)	171	1,921	890.2	3.30 (2.49–4.37)	2.75 (2.07–3.65)	2.38 (1.78–3.18)	
Association between TG variability and overall survival							
Q1 (≤15.4) (N=844)	152	2,884	527.0	ref	ref	ref	0.722
Q2 (15.4–22.5) (N=838)	130	2,867	453.5	0.86 (0.68–1.09)	0.91 (0.72–1.15)	0.90 (0.71–1.15)	
Q3 (22.5–30.9) (N=845)	137	2,897	472.9	0.89 (0.71–1.12)	0.94 (0.75–1.19)	0.98 (0.78–1.24)	
Q4 (>30.9) (N=841)	128	2,819	454.0	0.85 (0.67–1.08)	0.98 (0.77–1.24)	1.02 (0.80–1.30)	

*Model 1 is the unadjusted model; Model 2 is adjusted for age, sex, BMI, and mean of lipid measurements (mean of total cholesterol, LDL, HDL, or TG, respectively); Model 3 is further adjusted for the presence of CKD, DM, and HTN, smoking status, alcohol consumption, and cancer type.

*CKD, chronic kidney disease; DM, diabetes mellitus; HDL, high-density lipoprotein; HTN, hypertension; LDL, low-density lipoprotein; TG, Triglyceride.

^a^P for trend was estimated in Model 3.

### 3.3 Sensitivity analysis and subgroup analysis

We conducted a sensitivity analysis for cancer patients who achieved NED during the study period to be censored at NED status. As shown in **Table 3**, Q2 (aHR: 1.38, 95% CI: 1.28–1.49), Q3 (aHR: 1.77, 95% CI: 1.64–1.9), and Q4 (aHR: 2.09, 95% CI: 1.95–2.25) of CV in TC were significantly associated with an increased risk of mortality compared to Q1 (**Table 3**). Furthermore, when the use of lipid-lowering agents was censored considering patients who received lipid-lowering agents during the study period, Q2 (aHR: 1.32, 95% CI: 1.23–1.41), Q3 (aHR: 1.67, 95% CI: 1.57–1.78), and Q4 (aHR: 1.97, 95% CI: 1.85–2.1) of CV in TC were all significantly linked with a higher risk of death compared to Q1 (**Table 4**). Furthermore, in subgroup analyses, heterogeneity was not observed in age, sex, presence or absence of DM and HTN, smoking status, or alcohol consumption, except for cancer type (**Supplementary Table 1**). Using GEE models, after controlling covariates, an increase of 1 mg/dL in absolute changes of total cholesterol from baseline was found to significantly increase mortality risk by 0.4%. (**Supplementary Table 2**).

**Table 3:** Sensitivity analysis according to NED status.

	Events	Person-years	Incidence Rate (per 10,000 py)	Model 1	Model 2	Model 3
Association between TC variability and overall survival						
Q1 (≤8.2) (N=10,632)	1154	28,195	409.3	ref	ref	ref
Q2 (8.2–11.8) (N=10,698)	1642	28,122	583.9	1.43 (1.33–1.54)	1.38 (1.28–1.49)	1.38 (1.28–1.49)
Q3 (11.8–16.4) (N=10,623)	2223	27,676	803.2	1.98 (1.84–2.12)	1.81 (1.69–1.94)	1.77 (1.64–1.9)
Q4 (>16.4) (N=10,586)	2874	27,192	1,056.9	2.61 (2.44–2.8)	2.11 (1.97–2.26)	2.09 (1.95–2.25)

*Model 1 is the unadjusted model; Model 2 is adjusted for age, sex, BMI, and mean of total cholesterol; Model 3 is further adjusted for the presence of CKD, DM, and HTN, smoking status, alcohol consumption, and cancer type.

*CKD, chronic kidney disease; DM, diabetes mellitus; HTN, hypertension; NED, No evidence of disease.

**Table 4:** Sensitivity analysis according to the administration of lipid-lowering agents during the follow-up study period.

	Events	Person-years	Incidence Rate (per 10,000 py)	Model 1	Model 2	Model 3
Association between TC variability and overall survival						
Q1 (≤8.2) (N=10,632)	1497	54,847	272.9	ref	ref	ref
Q2 (8.2–11.8) (N=10,698)	2061	54,977	374.9	1.38 (1.29–1.47)	1.33 (1.24–1.42)	1.32 (1.23–1.41)
Q3 (11.8–16.4) (N=10,623)	2708	52,684	514.0	1.89 (1.77–2.01)	1.71 (1.6–1.82)	1.67 (1.57–1.78)
Q4 (>16.4) (N=10,586)	3533	50,715	696.6	2.56 (2.41–2.72)	2 (1.88–2.13)	1.97 (1.85–2.1)

*Model 1 is the unadjusted model; Model 2 is adjusted for age, sex, BMI, and mean of total cholesterol; Model 3 is further adjusted for the presence of CKD, DM, and HTN, smoking status, alcohol consumption, and cancer type.

*CKD, chronic kidney disease; DM, diabetes mellitus; HTN, hypertension.

## 4 Discussion

In this large-scale longitudinal study comprising 213,223 person-years, we found that a high CV quartile in TC was significantly associated with all-cause mortality after considering potential confounding variables. Despite considering NED status and the administration of lipid-lowering medications during the follow-up study period, our study’s findings were consistent. Additionally, we investigated the relationships between the CV quartiles of LDL-C, HDL-C, and TG and mortality risk. We found a significant relationship between the high CV quartiles of LDL-C and HDL-C and mortality risk, but not TG. We demonstrated the comprehensive prognostic role of lipid variability on mortality risk in cancer patients not receiving lipid-lowering medication.

In a systematic review and meta-analysis of the association of lipid variability with all-cause mortality, a high CV of TC, LDL-C, and HDL-C was significantly associated with all-cause mortality (6, 19). In our study, the association between the CV quartiles of TC, LDL, and HDL and mortality risk was significant but not for TG. In the two previous studies that examined the effect of the CV quartile of TC on all-cause mortality, the adjusted hazard ratio in Q4 was 1.26 (95% CI: 1.24–1.28) and 1.21 (95% CI: 1.05–1.40) compared to Q1 (7, 12). Two previous studies evaluated the effect of cholesterol fluctuations on mortality regardless of the administration of lipid-lowering agents. However, our large cohort study evaluated the effect in cancer patients who did not receive lipid-lowering agents, considering the possible effect of lipid-lowering agents on cholesterol fluctuations. Although previous studies have been conducted in different populations and settings from ours, our study found that the aHR in Q4 was 1.96 (95% CI: 1.84–2.08) compared to Q1 in cancer patients not receiving lipid-lowering medications. One previous study estimated that the aHR for all-cause mortality was 1.41 (95% CI: 1.36–1.45) for the low-mean/high variability of HDL-C compared with the high-mean/low-variability (8). In our study, the aHR of the highest variability of HDL (Q4) compared with the lowest variability (Q1) was 2.38 (95% CI: 1.78–3.18). In subgroup analysis, it was estimated that the HR of CV from TC to death was different for each cancer type (*P* for interaction<0.001). In subsequent studies, a larger sample size may be required to determine which cancer species are more vulnerable.

Several possible mechanisms have been implicated in the pathophysiology underlying the effect of lipid variability on the mortality risk of cancer patients not receiving lipid-lowering agents. High lipid variability may exacerbate the fluctuation of atherosclerotic plaque components, resulting in recurrent cholesterol crystallization and dissolution within the restricted region, compromising plaque stability and causing plaque rupture (10, 19, 20). Additionally, variations in cholesterol levels may lead to endothelial dysfunction (21–23). In a mouse study, endothelial inflammation increased endothelial permeability, and alteration of the glycocalyx was significant in early cancer metastatic events (24). Furthermore, dysfunctionally activated endothelial cells induced both the spontaneous metastasis of lung cancers in mice and pro-inflammatory signaling (25). In addition, dysregulated cholesterol homeostasis may affect cancer pathogenesis and metastasis by favoring cells resistant to ferroptotic cell death (26). Previous studies found that people with arteriosclerosis were twice as likely to develop cancer and die than those without arteriosclerosis and cancer patients were roughly twice as likely to die from arteriosclerosis than the general population (27, 28). Also, increased mortality risk is associated with high HDL-C variability, which may be a consequence of impaired cholesterol efflux from macrophages and peripheral organs, systemic diseases, and overall frailty (8, 29–31). Higher LDL-C variability has been associated with higher urine protein-to-creatinine ratio in chronic kidney disease patients and increased risk of atrial fibrillation (6, 32, 33). Additionally, higher LDL-C variability was associated with reduced cerebral blood flow and increased white matter hyperintensity burden (34, 35). Reduced cognitive function is linked to a slightly higher probability of death from cancer, along with a much higher risk of death from any cause (36).

The strength of our study is that it is the first to evaluate the association between the variability of lipid measurement and all-cause mortality with long-term follow-up in a large sample size of newly diagnosed cancer patients not receiving lipid-lowering agents. Second, we evaluated the effect of TC variability and LDL-C, TG, and HDL-C fluctuation on all-cause mortality. We conducted the study by limiting it to patients who did not take lipid-lowering agents to exclude the possible effects of lipid-lowering agents on lipid fluctuations. Third, we conducted two sensitivity analyses considering NED status and taking lipid-lowering agents during the study follow-up. Consequently, the main result of our study is consistent with sensitivity analysis. Finally, the results of the study may have clinical implications. Laboratory blood tests commonly conducted for cancer patients allow for easy measurement of lipid variability. Monitoring lipid variability when cancer is first diagnosed may help to lower mortality rates. In addition, if cancer patients exhibit high lipid variability in TC, LDL-C, and HDL-C, physicians can educate them about the importance of controlling and maintaining cholesterol levels through lifestyle modifications or medication to prevent fluctuations. However, our study has several limitations. First, since the study relies on a sample of Korean people in a single center, our findings cannot be extrapolated to people of different ethnicities. In addition, because only those with at least three lipid measurements performed at the hospital within 2 years of their cancer diagnosis were included in the study, there may be a selection and surveillance bias in that those who visited the hospital more frequently were included in the study. Additionally, unmeasured, or residual confounding, such as cancer therapy, eating habits, physical activity, or specific cancer treatment details could not be completely eliminated, although the multivariable analysis considered many confounders. Third, while our study explored association between cholesterol fluctuations and mortality in cancer patients, a subsequent study is necessary to study the biological mechanisms related to specific cancer types, alterations in endothelial function, or inflammation. Finally, since our hospital’s medical electronic records and self-questionnaires were used when another hospital prescribed the drug, it could be missed in the analysis. In this large-scale longitudinal investigation, we found that after controlling for any potential confounding variables, the high CV quartile in TC was significantly associated with all-cause death in newly diagnosed cancer patients.

## 5 Conclusions

Increasing mortality risk was significantly associated with high CV quartiles of LDL-C and HDL-C but not TG. These findings suggest that lipid fluctuation can be a comprehensive prognostic marker of mortality risk in cancer patients not receiving lipid-lowering drugs.

## Data availability statement

The original contributions presented in the study are included in the article/**Supplementary Material**. Further inquiries can be directed to the corresponding author.

## Ethics statement

The studies involving humans were approved by The Institutional Review Board (IRB) of Samsung Medical Center approved this study (approval no. SMC 2023-01-085). An informed consent exemption was granted by the IRB because all data provided by the CDW of SMC to researchers were de-identified. The studies were conducted in accordance with the local legislation and institutional requirements. The ethics committee/institutional review board waived the requirement of written informed consent for participation from the participants or the participants’ legal guardians/next of kin because The Institutional Review Board (IRB) of Samsung Medical Center approved this study (approval no. SMC 2023-01-085). An informed consent exemption was granted by the IRB because all data provided by the CDW of SMC to researchers were de-identified.

## Author contributions

SK: Conceptualization, Data curation, Investigation, Software, Writing – original draft. GK: Conceptualization, Writing – original draft, Writing – review & editing. SC: Writing – review & editing. RO: Writing – review & editing. JYK: Writing – review & editing. YBL: Writing – review & editing. S-MJ: Writing – review & editing. KH: Writing – review & editing. JHK: Conceptualization, Investigation, Methodology, Project administration, Supervision, Writing – review & editing, Writing – original draft.
