# Supplementary material for: Association between lipid variability and the risk of mortality in cancer patients not receiving lipid-lowering agents
Source: Front Oncol. 2023 Oct 5;13:1254339. doi: 10.3389/fonc.2023.1254339 (PMC10586791; doi:10.3389/fonc.2023.1254339)
Supplement: Supplementary file 1 [file Table_1.docx]

**Supplementary Table 1. Subgroup analysis**

|  | Q4 (vs. Q1~3) (aHR, 95% CI) | *P* for interaction |
| --- | --- | --- |
| **Age** |  | 0.143 |
| <60 (N=23,191) | 1.53(1.43–1.64) |  |
| ≥60 (N=19,348) | 1.41(1.34–1.49) |  |
| **Sex** |  | 0.269 |
| Female (N=19,299) | 1.4(1.29–1.52) |  |
| Male (N=23,240) | 1.47(1.4–1.55) |  |
| **BMI** |  | 0.799 |
| <25 (N=30,819) | 1.4(1.34–1.47) |  |
| ≥25 (N=11,720) | 1.5(1.36–1.65) |  |
| **DM** |  | 0.125 |
| No (N=33,937) | 1.47(1.39–1.54) |  |
| Yes (N=8,602) | 1.41(1.31–1.52) |  |
| **HTN** |  | 0.812 |
| No (N=25,221) | 1.45(1.37–1.55) |  |
| Yes (N=17,318) | 1.45(1.37–1.54) |  |
| **Smoking Status** |  | 0.523 |
| Never (N=26,789) | 1.46(1.37–1.55) |  |
| Ever (N=7,894) | 1.4(1.28–1.52) |  |
| Current (N=7,856) | 1.44(1.33–1.57) |  |
| **Alcohol consumption** |  | 0.154 |
| Never (N=24,048) | 1.48(1.4–1.57) |  |
| Ever (N=11,216) | 1.46(1.36–1.57) |  |
| Current (N=7,275) | 1.29(1.16–1.45) |  |
| **Cancer types** |  | <0.001 |
| Gastrointestinal (N=18,281) | 1.27(1.19–1.35) |  |
| Urology (N=961) | 1.59(1.2–2.12) |  |
| Gynecology (N=111) | 1.96(0.88–4.38) |  |
| Breast (N=7,526) | 1.46(1.17–1.82) |  |
| Hepato-Pancreatobiliary (N=4,576) | 1.41(1.29–1.54) |  |
| Lung (N=7,910) | 1.56(1.43–1.7) |  |
| Thyroid (N=982) | 2.97(1.6–5.51) |  |
| Others (N=2,192) | 1.87(1.59–2.2) |  |

*Abbreviations: BMI=body mass index, CKD=chronic kidney disease, DM=diabetes mellitus, HTN=hypertension

**Supplementary Table 2. Results of Generalized Estimating Equations (GEE) model for association between absolute changes of repeated lipid measurements and death**

|  | **Baseline-6 months**  (N=36,790) | **Baseline-12 months**  **(N=30,105)** | **Baseline-18 months**  **(N=23,885)** | **Baseline-24 months**  **(N=11,565)** | **Model 1**  **(OR, 95% CI)** | **Model 2**  **(OR, 95% CI)** |
| --- | --- | --- | --- | --- | --- | --- |
| **Changes in total cholesterol from baseline, mg/dL** | 29.1 ± 23.2 | 29.2 ± 23.3 | 29.7 ± 23.8 | 30.3 ± 24.2 | 1.006  (1.005, 1.007) | 1.004  (1.004, 1.005) |

*Model 1 is the unadjusted model; Model 2 is adjusted for age, sex, BMI, the presence of CKD, DM, and HTN, smoking status, alcohol consumption, and cancer type

*Abbreviations: BMI=body mass index, CI=confidence interval, CKD=chronic kidney disease, DM=diabetes mellitus, HDL=high-density lipoprotein, HTN=hypertension, LDL=low-density lipoprotein, OR=Odds Ratio, TG=Triglyceride
